# Inhibition of *Staphylococcus aureus* biofilm by *Lactobacillus* isolated from fine cocoa

**DOI:** 10.1186/s12866-016-0871-8

**Published:** 2016-10-28

**Authors:** Tauá Alves Melo, Thalis Ferreira dos Santos, Milena Evangelista de Almeida, Luiz Alberto Gusmão Fontes Junior, Ewerton Ferraz Andrade, Rachel Passos Rezende, Lucas Miranda Marques, Carla Cristina Romano

**Affiliations:** 1 Department of Biological Sciences, Santa Cruz State University, Ilhéus-Itabuna Road, km 16 Salobrinho, Ilhéus, 45662-900 Bahia State Brazil; 2 Multidisciplinary Institute for Health, Federal University of Bahia, Rio de Contas Street, Candeiasn 17,58 Block, Vitória da Conquista, 45029-094 Bahia State Brazil

**Keywords:** Antistaphylococcal, Biofilm, *ica*, *Lactobacillus fermentum*, Probiotic properties

## Abstract

**Background:**

Biofilm production represents an important virulence and pathogenesis factor for *Staphylococcus aureus*. The formation of biofilms on medical devices is a major concern in hospital environments, as they can become a constant source of infection. Probiotic bacteria, such as *Lactobacillus fermentum* and *L. plantarum*, have been found to inhibit biofilm formation; however little is known about the underlying mechanism. In this study, we tested the activity of supernatants produced by *L. fermentum* TCUESC01 and *L. plantarum* TCUESC02, isolated during the fermentation of fine cocoa, against *S. aureus* CCMB262 biofilm production. We measured inhibition of biofilm formation in vitro and analyzed biofilm structure by confocal and electronic microscopy. Additionally, we quantified the expression of *S. aureus* genes *icaA* and *icaR* involved in the synthesis of the biofilm matrix by real-time PCR.

**Results:**

Both *Lactobacillus* supernatants inhibited *S. aureus* growth. However, only *L. fermentum* TCUESC01 significantly reduced the thickness of the biofilm, from 14 μm to 2.83 μm (at 18 mg∙mL^−1^, 90 % of the minimum inhibitory concentration, MIC), 3.12 μm (at 14 mg∙mL^−1^, 70 % of the MIC), and 5.21 μm (at 10 mg∙mL^−1^, 50 % of the MIC). Additionally, *L. fermentum* TCUESC01 supernatant modulated the expression of *icaA* and *icaR*.

**Conclusions:**

*L. fermentum* TCUESC01 reduces the formation of *S. aureus* biofilm under subinhibitory conditions. Inhibition of biofilm production probably depends on modulation of the *ica* operon.

## Background

*Staphylococcus aureus* is a widely distributed and opportunistic human pathogen. It is the causative agent of both simple skin infections and potentially life-threatening systemic complications such as toxic shock syndrome [1]. *S. aureus* is a major concern in hospital environments because asymptomatic carriers represent a great risk factor to patients subjected to long hospitalization periods [2].

Some aspects of *S. aureus* pathogenicity are related to biofilm production, which increases resistance to chemotherapeutic treatments and to the host’s defense mechanisms [3]. The biofilm is composed of cells adhering to a surface and held together by a polymer matrix. The main components of the matrix are polysaccharides, proteins, and extracellular DNA (eDNA) [4].

*S. aureus* secretes polysaccharides of intercellular adhesion (PIAs), which are composed mostly of β-1, 6-*N*-acetylglucosamine residues. PIA production and excretion is controlled by the *icaADBC* operon [5]. *icaA*, the first gene to be transcribed, leads to production of short chains of *N*-acetylglucosamine oligomers [6]. i*caD*, co-expressed with *icaA*, enhances oligomer production by about 20 fold [7, 8]. *icaC* is responsible for increasing oligomer chains and possibly for translocation to the cell surface [8, 9]. Finally, *icaB* is thought to deacetylate poly-*N*- acetylglucosamines [9]. The *icaADBC* locus is regulated by a transcriptional repressor located upstream encoded by *icaR* [10]. This repressor protein can bind to the *ica operon* promoter region close to the *icaA* start codon [8]. Additional factors that can negatively influence the *ica* operon in *S. aureus* include expression of the weak repressor *TcaR*, global regulation by *SarA*, and the insertion sequence element IS256 in the *ica* locus [8, 11]. The *ica* operon is also regulated by environmental factors, which play an important role in the response to anaerobic growth (*SrrAB*), in the response to stress (*Spx*), supplementation with glucose or ethanol, osmolarity, temperature, and low concentrations of antibiotics [8, 11, 12]. Another way of controlling this operon is through a regulatory protein responsible for biofilm formation, *Rbf*, which can repress transcription of *IcaR* and indirectly increase expression of the gene *icaADBC* [11].

*S. aureus* biofilms are commonly treated with antibiotics, such as protein synthesis inhibitors that target the cell membrane and cell wall, as well as inhibitors of DNA and RNA synthesis [4], or antimicrobials like Cu^2+^ that lead to cell membrane breakage and subsequent cell lysis [13]. The indiscriminate use of antibiotics for the treatment of bacterial infections has been suggested to be responsible for the appearance of multidrug-resistant bacteria such as methicillin-resistant *S. aureus* strains [14]. In this context, alternatives to antibiotic therapy are more than welcome. Probiotics represent a possible option, as the microorganisms that produce them have been proven effective in the prevention and control of human pathogens [15].

*Lactobacillus fermentum* TCUESC01 and *L. plantarum* TCUESC02 strains have been recently isolated from the fermentation of fine cocoa seeds. Our group has demonstrated their anti-inflammatory potential and technological properties. We observed that these strains exhibited probiotic characteristics in vivo in an experimental colitis model, reducing histological damage and the systemic concentration of inflammatory cytokines. Additionally, they inhibited the growth of pathogenic bacteria and displayed high resistance to the stressful conditions of the gastrointestinal tract and industrial environments (unpublished observations). In this study, we evaluated the activity of cell-free supernatant from *L. fermentum* TCUESC01 and *L. plantarum* TCUESC02 on *S. aureus* CCMB262 biofilm formation.

## Methods

### Microorganisms and culture conditions

Pure cultures of *L. fermentum* TCUESC01, *L. plantarum* TCUESC02, and *S. aureus* CCMB262 were used. TCUESC01 and TCUESC02 strains were previously isolated by our group during fine cocoa fermentation [16]. The cultures were stored at −80 °C in 10 % skim milk (Molico®, Nestlé, São Paulo Brazil) with 30 % glycerol. Lactobacilli were cultured in de Man, Rogosa, and Sharpe (MRS) broth (1 % peptone, 0.8 % meat extract, 0.4 % yeast extract, 2 % glucose, 0.5 % sodium acetate, 0.2 % dipotassium hydrogen phosphate, 0.02 % magnesium sulfate eptahydrate, 0.005 % manganese sulfate tetrahydrate, 0.02 % triammonium citrate) (HiMedia®, Mumbai, India), for 48 h at 37 °C. Species identity was confirmed by *16S rDNA* sequencing and strains were deposited in the GenBank database [17] under accession numbers KU244478 and KU244476.

*S. aureus* CCMB262 was obtained from the Microorganisms Culture Collection of Bahia (CCMB), Brazil. The strain, which is resistant to streptomycin and dihydrostreptomycin, was cultured in tryptic soy broth (TSB; DIFCO, Becton Dickinson, Franklin Lakes, NJ, USA) supplemented with 1 % (w/v) glucose (Glc) at 37 °C with agitation (250 rpm) for 18 h. For inoculum standardization, *S. aureus* cells were homogenized in saline solution (NaCl 0.85 %) and the suspension was diluted to 0.5 × 10^8^ CFU∙mL^−1^ using a spectrophotometer (Evolution 60, Thermo Fisher Scientific, Waltham, MA, USA).

### Supernatant preparation and lyophilization process

*L. fermentum* TCUESC01 and *L. plantarum* TCUESC02 were cultured in 30 mL of MRS broth. Following centrifugation at 10,000 × *g* for 15 min at 4 °C, culture supernatants were collected and filtered through a 0.22 μm nitrocellulose membrane. Supernatants and samples containing medium culture only (control) were frozen at −80 °C for 24 h and lyophilized (Lyophilizer LS3000, Terroni, São Carlos, Brazil). Following lyophilization, the samples were weighed and stored at −20 °C. They were then rehydrated with sterile deionized water prior to use.

### Minimum inhibitory concentration (MIC) assay

MIC assays were performed by microdilution in 96-well plates (Costar®, Corning, NY, USA), in accordance with recommendations from the Clinical and Laboratory Standards Institute [18]. A serial dilution was performed starting with 40 μg∙mL^−1^ of TCUESC01 and TCUESC02 supernatants on Mueller Hinton (MH) medium containing 5 × 10^5^ CFU∙mL^−1^ of *S. aureus* CCMB262 per well. The same procedure was performed with the following controls: lyophilized medium without *Lactobacillus* (MRS control); MH without inoculum (medium sterility control - MC); MH containing 5 × 10^5^ CFU∙mL^−1^ of *S. aureus* CCMB262 (positive control); MH containing 5 × 10^5^ CFU∙mL^−1^ of *S. aureus* CCMB262 and 12.5 μg∙mL^−1^ chloramphenicol (negative control). The microplate was incubated for 24 h at 37 °C and revealed with 20 μL of Resazurin (0.01 %) for 30 min at 37 °C. At the same time, 5 μL of each suspension (samples and control) was cultured on MH agar (MHA; DIFCO) for 24 h at 37 °C, after which the inhibitory concentrations were classified as bactericidal or bacteriostatic. The entire experiment was performed three times with three independent repetitions.

### Preparation of subinhibitory concentrations of TCUESC01 and TCUESC02 supernatants

To avoid killing all *S. aureus*, lyophilized supernatants of the two *Lactobacillus* strains were weighed and diluted to concentrations below the MIC. Thus, dilutions were made using subinhibitory concentrations of 90, 70, and 50 % of MIC.

### Biofilm formation on a polystyrene plate

The ability of *S. aureus* to form biofilms following treatment with *Lactobacillus* supernatant was analyzed according to the methodology proposed by Oliveira et al. (2014) [19]. Briefly, *S. aureus* CCMB262 was cultured in 5 mL TSB with 1 % Glc for 18 h at 37 °C under agitation (250 rpm) and treated with *Lactobacillus* supernatants (90 %, 70 %, or 50 % of the MIC) or control medium. The cultures were diluted (1:100) in the same medium, 200 μL was inoculated in a 96-well plate (Costar®), and plates were incubated at 37 °C for 24 h. The plates were washed twice with phosphate-buffered saline (PBS), dried for 1 h at 65 °C, 1 % crystal violet was added, and the plates were incubated for a further 30 min at 25 °C. Each well was washed twice with PBS and 200 μL PBS was added prior to measuring absorbance at 492 nm (A_492nm_) using a microplate reader (VersaMax; Molecular Devices®, Sunnyvale, CA, USA). The experiment was carried out in quadruplicate with at least two independent experiments. Biofilm production was compared to that of *Streptococcus pyogenes* ATCC75194 (A_492nm_ = 0.07). The biofilm formation index (BFI) was calculated as follows:

$$ BFI=\frac{x}{y} $$

where x is the optical density at A_492nm_ of the biofilm and y is the optical density at A_492nm_ of *Streptococcus pyogenes* (0.07).

Based on the BFI, *S. aureus* cultures were classified as non-producers (less than or equal to zero), weak producers (less than 1), moderate producers (between 1 and 2), producers (between 2 and 3), or strong biofilm producers (greater than 4).

### Biofilm evaluation by confocal laser scanning microscopy

*S. aureus* CCMB262 biofilm viability was assessed according to Hobby et al. (2012) with some modifications [20]. *S. aureus* CCMB262 was cultured in TSB with 1 % Glc for 18 h at 37 °C in 12-well culture plates (Costar®) containing 18 mm coverslips. Cells were cultured in the presence of *L. fermentum* TCUESC01 (90, 70, and 50 % of MIC) and *L. plantarum* TCUESC02 (90 and 70 % of MIC) supernatants or control medium. Microplates were incubated for 18 h at 37 °C, then coverslips were washed twice with 0.85 % NaCl and stained with 0.3 μg∙mL^−1^ 4′, 6′-diamidino-2-phenylindole (DAPI; Molecular Probes, Carlsbad, CA, USA) and 2.5 μg∙mL^−1^ propidium iodide (PI; Invitrogen, Carlsbad, CA, USA) for 15 min in the dark. The coverslips were observed with a confocal laser scanning microscope (Carl Zeiss LSM 700; Jena, Germany), equipped with an argon laser at 488 nm and two helium/neon lasers at 543 nm.

### Cultivation of *S. aureus* CCMB262 in the presence or absence of *L. fermentum* TCUESC01 supernatant at a subinhibitory dose (50 % of MIC)

To confirm that the concentration used to inhibit the biofilm was not lethal to *S. aureus*, a growth curve was constructed. *S. aureus* (75 μL, 0.5 × 10^8^ CFU∙mL^−1^) was inoculated in 75 mL TSB with 1 % Glc and cultivated in the presence or absence of 750 mg of TCUESC01 supernatant (50 % of MIC) for 24 h at 37 °C under agitation (250 rpm). Aliquots were collected every 4 h to record optical density at 660 nm and count CFU∙mL^−1^ in mannitol salt agar.

### Phenotypic evaluation of biofilms by scanning electron microscopy (SEM)

The phenotype of *S. aureus* CCMB262 biofilm was evaluated by SEM according to Pitino et al. (2012) with some modifications [21]. *S. aureus* was cultured in TSB with 1 % Glc in the presence or absence of TCUESC01 supernatant in 12-well culture plates (Costar®) containing 18 mm coverslips. After incubation for 24 h at 37 °C, the coverslips were washed twice with 0.85 % NaCl and dehydrated with 50, 60, 70, 80, and 90 % acetone for 10 min each, and twice with 100 % acetone for 10 min. The coverslips were subsequently dehydrated in a critical point dryer CPD 030 (BAL-TEC®, Balzers, Germany) using liquid carbon dioxide as transition fluid. The samples were placed in aluminum pieces, metallized with a SCD-050 (BAL-TEC®, Alzenau, Germany), and viewed under a scanning electron microscope (Quanta 250, FEI, Hillsboro, OR, USA).

### Total RNA extraction

*S. aureus* CCMB262 was cultured in TSB with 1 % Glc for 18 h at 37 °C under agitation (250 rpm), in the presence or absence of *L. fermentum* TCUESC01 supernatant (50 % of the MIC). The samples were then diluted in the same medium (1:100) and incubated at 37 °C for another 18 h, after which aliquots were collected and used immediately for RNA extraction.

Each sample (1 mL) was centrifuged at 10,000 × *g* for 10 min at 4 °C. The supernatant was discarded and RNA from the pellet was extracted with the RNAqueous® Phenol-free total RNA isolation kit (Ambion®, Waltham, MA, USA), according to the manufacturer’s recommendations. The RNA was visualized on a 1 % agarose gel and quantified with a NanoDrop 2000 spectrophotometer (Thermo Fisher Scientific).

### Reverse transcription, PCR, and real-time PCR

cDNA was synthesized using 25 mg RNA and the SuperScript™ First-Strand Synthesis System for RT-PCR kit (Invitrogen) following the manufacturer’s protocol. Specific primers (Invitrogen) were selected according to Yu et al. (2012) [22]: *icaR* Forward (5′-ATCTAATACGCCTGAGGA-3′), Reverse (5′-TTCTTCCACTGCTCCAA-3′); *icaA* Forward (5′-TTTCGGGTGTCTTCACTCTAT-3′), Reverse (5′-CGTAGTAATACTTCGTGTCCC-3′); *16S rRNA S. aureus* Forward (5′-CGTGGAGGGTCATTGGA-3′), Reverse (5′-CGTTTACGGCGTGGACT-3′). Amplification was carried out in a Mastercyler Gradient Nexus Thermal Cycler (Eppendorf, Hauppage, NY, USA). The cDNA was then quantified with a NanoDrop 2000 spectrophotometer (Thermo Fisher Scientific).

Gene expression analysis was carried out on a 7500 Fast real-time PCR system (Applied Biosytems, Waltham, MA, USA), using 10 ng cDNA and the KAPA SYBR® qPCR Master Mix ABI Prism™ kit (Kapa Biosystems, Wilmington, MA, USA) according to the manufacturer’s recommendations. The PCR reaction was carried out in quintuplicate with an initial denaturation at 95 °C for 5 min followed by 40 cycles of amplification at 95 °C for 20 s, 60 °C for 20 s, and 72 °C for 20 s. Data were normalized to*16S rRNA* (endogenous control) and the relative quantification (RQ) was calculated with 7500 Software v 2.3 (Applied Biosytems).

### Statistical analysis

Average, standard deviation, *t*-test, analysis of variance, Tukey’s multiple comparison test, graphics, and other statistical analyses were performed using the Graphpad® prism 5.0 software (GraphPad Software, San Diego, CA, USA).

## Results and discussion

**Fig. 1:**
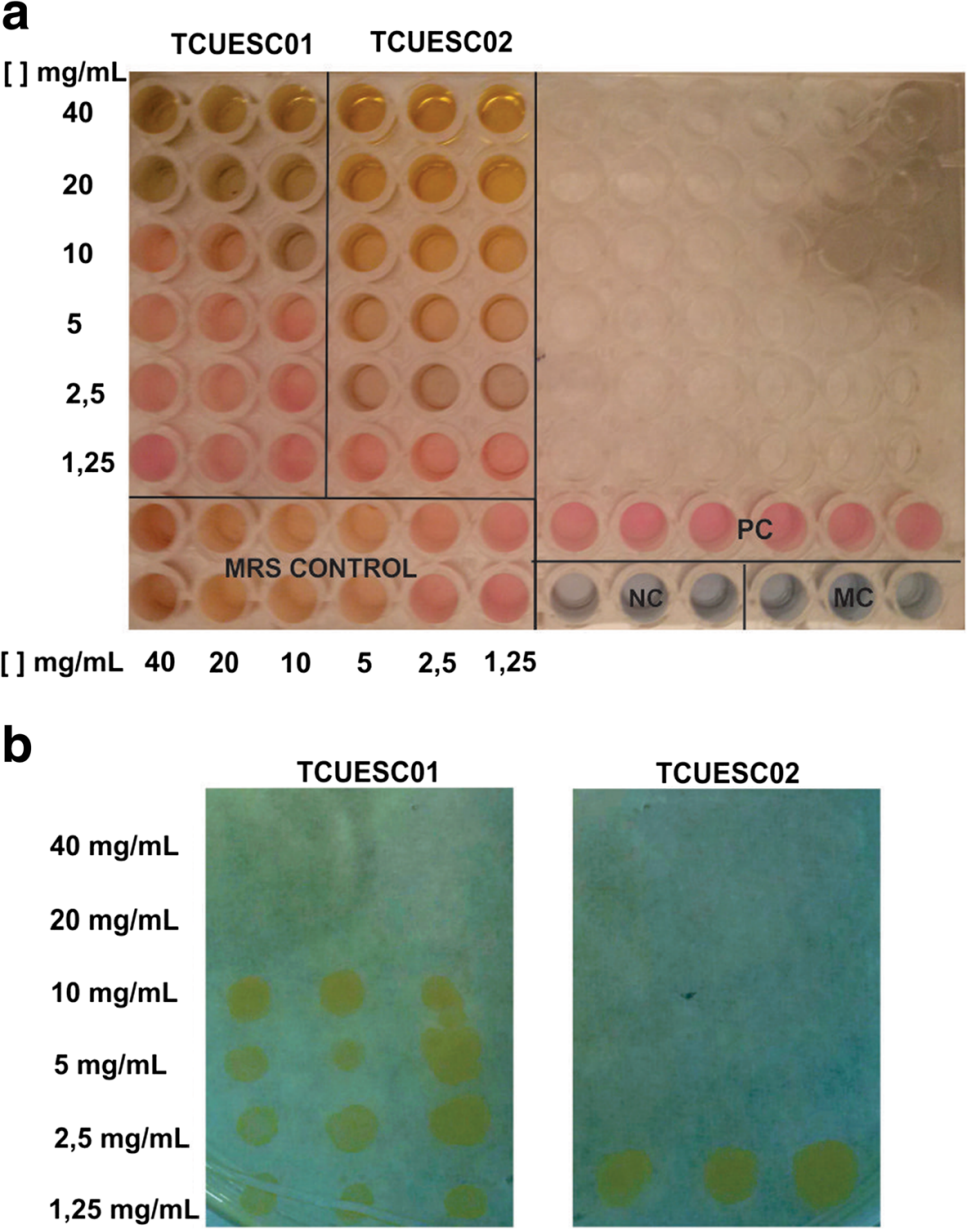
**a** Effect of minimum inhibitory concentration (MIC) of *Lactobacillus fermentum* TCUESC01 and *L. plantarum* TCUESC02 supernatants on *Staphylococcus aureus* CCMB262 growth. MRS Control, MRS medium without lyophilized *Lactobacillus* cells; PC, positive control for *S. aureus*; NC, negative control, *S. aureus* with 12.5 μg∙mL^−1^ chloramphenicol; MC, sterility control of MH medium. **b** Bactericidal/bacteriostatic activity of *L. fermentum* TCUESC01 and *L. plantarum* TCUESC02 supernatants (at MIC doses) against *S. aureus* CCMB262 grown on MHA

Antimicrobial susceptibility tests revealed that the supernatant from *L. plantarum* TCUESC02 had a stronger inhibitory effect on *S. aureus* CCMB262 growth (MIC, 2.5 mg∙mL^−1^) than that of *L. fermentum* TCUESC01 (MIC, 20 mg∙mL^−1^) (Fig. 1). The potential of *Lactobacillus* species to inhibit pathogens of clinical importance such as *S. aureus* had been evaluated before. Accordingly, Al Kassaa et al. (2014) showed that *L. fermentum* CMUL054 and *L. plantarum* CMUL140 were active against *S. aureus* ATCC33862 [23]. Moreover, Hor and Liong (2014) analyzed 87 lactic acid bacteria strains and three strains of bifidobacteria, and found that all strains inhibited the growth of *S. aureus* by 0.5 to 34.2 %; *L. fermentum* and *L. plantarum*, which were isolated from milk, inhibited growth by around 20 % [24]. Although inhibition of *S. aureus* by lactic bacteria has been reported in many studies [23–26], few of them have calculated the MIC from extracellular bacterial extracts. This has made it difficult to assess the extracts’ inhibitory action. Lactobacilli produce various secondary metabolites that exhibit antimicrobial activity, such as organic acids, ethyl alcohol, bacteriocins, hydrogen peroxide, and surfactants [15, 26–28]. The strains used in this study were isolated during cocoa fermentation, a process characterized by high temperature conditions, low oxygen concentrations, and low glucose availability. Lactobacilli are probably selected for their ability to withstand these conditions, which makes them an interesting source of compounds with biological activities of human interest.

**Fig. 2:**
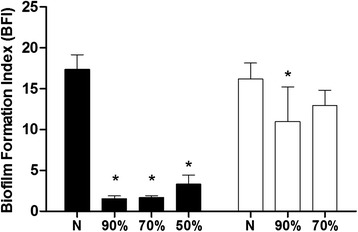
Biofilm formation index for *S. aureus* CCMB262 treated or not with TCUESC01 and TCUESC02 supernatants. ■, *L. fermentum* TCUESC01; □, *L. plantarum* TCUESC02; N, untreated control; 90, 70, and 50 %, MIC doses used for the treatment; *, statistical significance compared to the control (*p* < 0.001)

*S. aureus* CCMB262 is regarded as a strong biofilm producer (BFI 17.39). After treatment with *L. fermentum* TCUESC01 supernatant, we noticed a significant reduction in the BFI (*p* < 0.001). Upon treatment with 90 % (18 mg∙mL^−1^) and 70 % of the MIC (14 mg∙mL^−1^), classification of *S. aureus* CCMB262 changed from strong to moderate biofilm producers (BFI 1.55 and 1.69 respectively); it went from strong to biofilm producer (BFI 3.3) when treated with 50 % of the MIC (10 mg∙mL^−1^) (Fig. 2).

*L. plantarum* TCUESC02 was less effective against *S. aureus* CCMB262 biofilm formation (MIC, 2.5 mg∙mL^−1^). We found a significant difference in the BFI only when using 90 % of the MIC (*p* < 0.01), meaning that *S. aureus* CCMB262 was still a strong biofilm producer. We did not observe statistically significant differences between treatments with 90 % (2.25 mg∙mL^−1^) or 70 % of the MIC (1.75 mg∙mL^−1^) for this strain. In a previous report, the supernatant of *L. bulgaricus* FTDC8611 inhibited significantly *S. aureus* biofilm, an effect attributed to organic acids [24]. Also, Ait Ouali et al.(2014) demonstrated that *L. pentosus* LB3F2 had antimicrobial and antibiofilm activity against *S. aureus* SA3 [29].

**Fig. 3:**
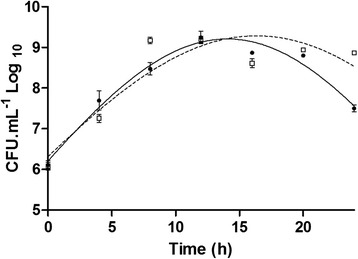
*S. aureus* CCMB262 growth in the presence or not of *L. fermentum* TCUESC01 supernatant at a subinhibitory dose (50 % of MIC).● and continuous line, standard growth curve with *S. aureus* CCMB262 (control); □ and dashed line, growth curve of *S. aureus* CCMB262 with addition of *L. fermentum* TCUESC01 supernatant at 50 % of the MIC (10 mg∙mL^−1^). The result represents the average of three experiments

A comparison between bacterial growth curves in the presence or absence of TCUESC01 supernatant at 50 % of the MIC (10 mg∙mL^−1^) revealed this concentration was not lethal to *S. aureus* CCMB262. This indicates that the decrease in biofilm formation was not caused by death of the pathogen (Fig. 3). Instead, TCUESC01 appeared to secrete a modulatory substance capable of interfering with the pathogen’s capacity to form biofilms.

**Fig. 4:**
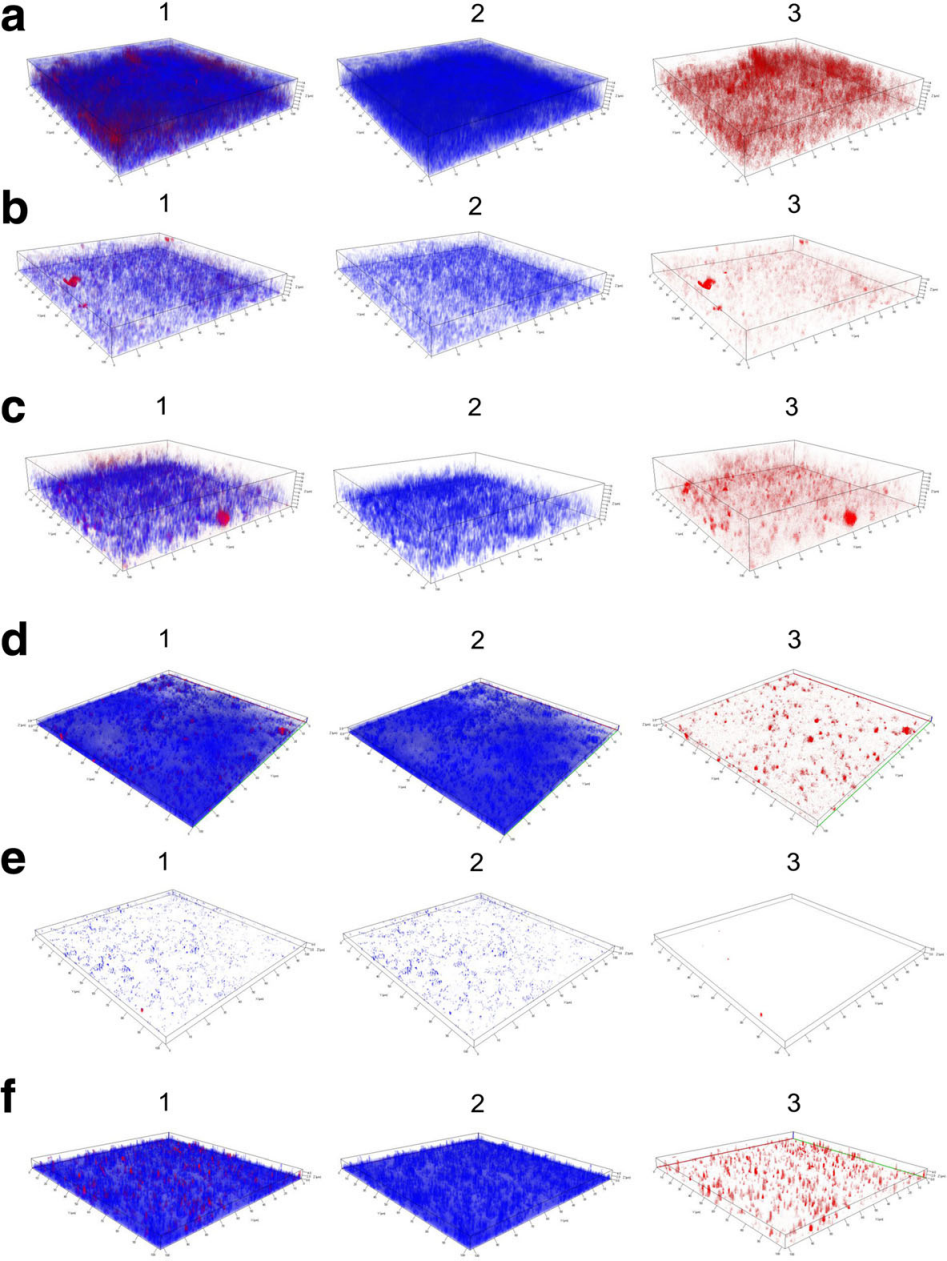
Confocal microscopy of *S. aureus* CCMB262 treated with *L. fermentum* TCUESC01 and *L. plantarum* TCUESC02 supernatants at subinhibitory doses. Treatments were as follows: **a** untreated *S. aureus* CCMB262; **b** TCUESC02 at 90 % of the MIC (2.25 mg∙mL^−1^); **c** TCUESC02 at 70 % of the MIC (1.75 mg∙mL^−1^); **d** TCUESC01 at 90 % of the MIC (18 mg∙mL^−1^); **e** TCUESC01 at 70 % of the MIC (14 mg∙mL^−1^); **f** TCUESC01 at 50 % of the MIC (10 mg∙mL^−1^). The biofilm was stained with DAPI (blue, panel 2) and PI (red, panel 3); panel 1 shows the overlapping of panels 2 and 3

**Fig. 5:**
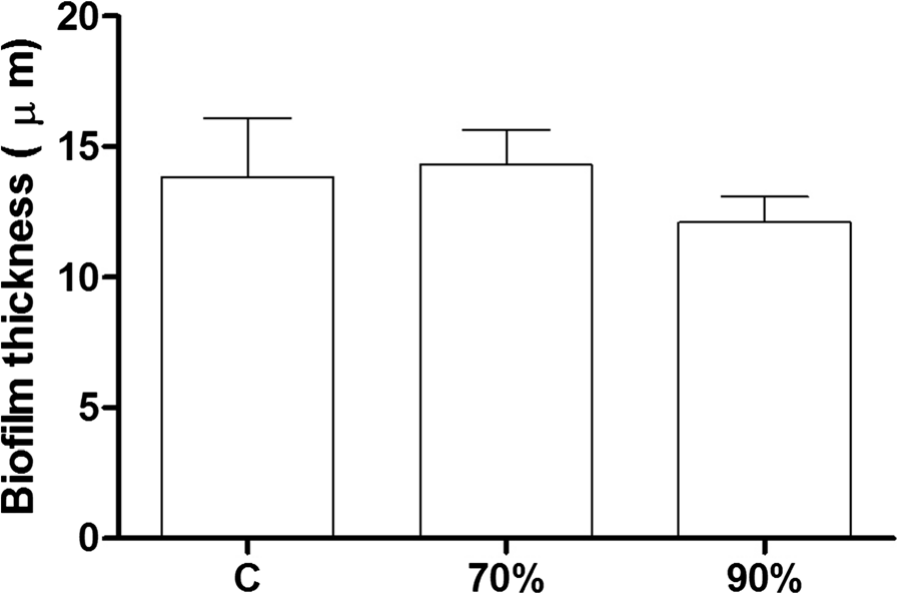
Thickness of *S. aureus* CCMB262 biofilm treated with *L. plantarum* TCUESC02 supernatant at subinhibitory doses. Treatments were as follows: C, untreated control; 70 %, 70 % of the MIC (1.75 mg∙mL^−1^); 90 %, 90 % of the MIC (2.25 mg∙mL^−1^). No significant difference could be observed

To confirm the results on biofilm inhibition, we visualized its structural organization by confocal microscopy (Fig. 4). To this end, we used two fluorescent nucleic acid stains, DAPI and PI. DAPI penetrates the membrane of viable cells, whereas PI cannot access intact cells and only stains dead cells. The biofilm of *S. aureus* CCMB262 presented an irregular distribution of living and dead cells, with dense clusters of eDNA/dead cells. Treatment with TCUESC01 supernatant (90, 70, and 50 % of the MIC) substantially reduced *S. aureus* biomass, with a proportional decrease in both living and dead cells. A reduced amount of eDNA and/or dead cells was observed also in the biofilm of *S. aureus* treated with TCUESC02 supernatant (Fig. 5). eDNA is one of the main components of biofilms [4]. It functions as a cohesive factor between bacteria, stabilizing the overall biofilm structure [30]. Moreover, it increases resistance to antimicrobials, such as aminoglycosides [31] and vancomycin [32]. Thus, reduction of eDNA production represents a potential tool for microbial control.

**Fig. 6:**
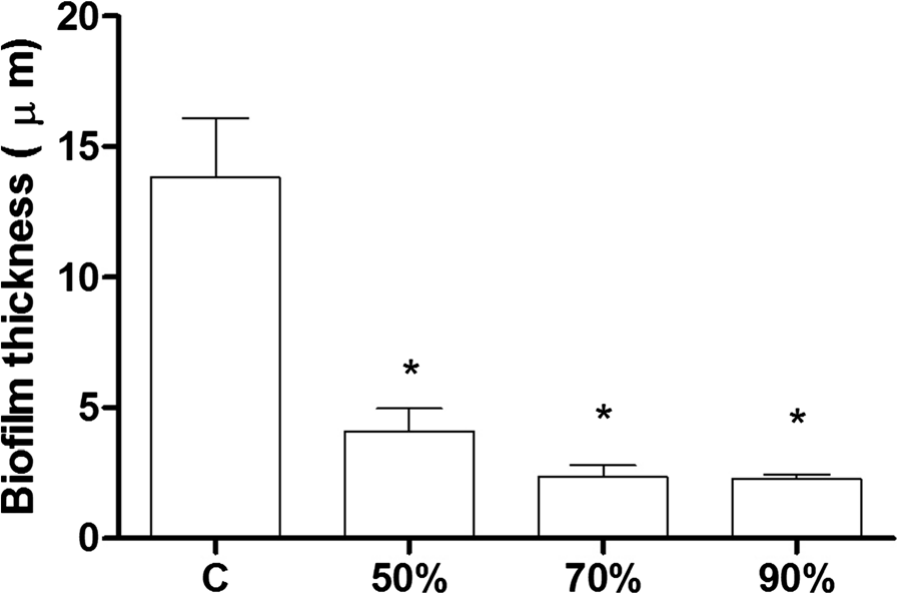
Thickness of *S. aureus* CCMB262 biofilm treated with *L. fermentum* TCUESC01 supernatant at subinhibitory doses. Treatments were as follows: C, untreated control; 50 %, 50 % of the MIC (10 mg∙mL^−1^); 70 %, 70 % of the MIC (14 mg∙mL^−1^); 90 %, 90 % of the MIC (18 mg∙mL^−1^).*, statistical significance at *p* < 0.05, analysis of variance

Microscopy analysis revealed also a significant reduction in *S. aureus* biolfilm thickness at all tested subinhibitory concentrations of *L. fermentum* TCUESC01 supernatant (*p* < 0.05). Whereas the average thickness of the biofilm in the control was 14 μm, samples treated with 90 % (18 mg∙mL^−1^), 70 % (14 mg∙mL^−1^), and 50 % (10 mg∙mL^−1^) of the MIC presented a thickness of 2.83, 3.12, and 5.21 μm, respectively (Fig. 6). It should be noted, that TCUESC02 did not cause any significant reduction in biofilm thickness.

**Fig. 7:**
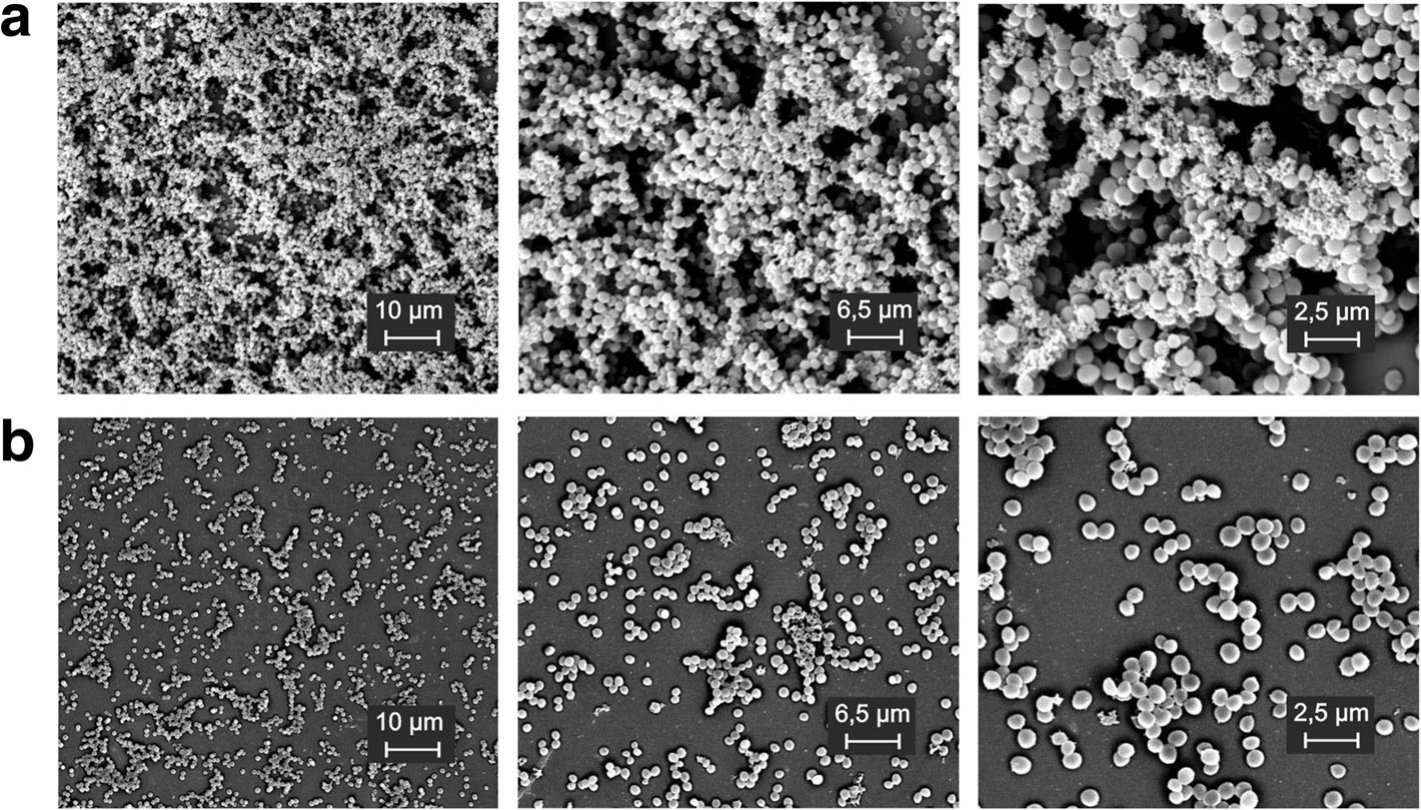
Scanning electron microscopy of *S. aureus* CCMB262 biofilm treated or not with *L. fermentum* TCUESC01 supernatant. Treatments were as follows: **a** untreated control; **b** 50 % of the MIC

To visualize the intimate structure of the *S. aureus* biofilm we used SEM. Following treatment with *L. fermentum* TCUESC01 supernatant (50 % of the MIC), we noticed a decrease in the biofilm matrix (Fig. 7), suggesting possible interference with the production of PIAs.

**Fig. 8:**
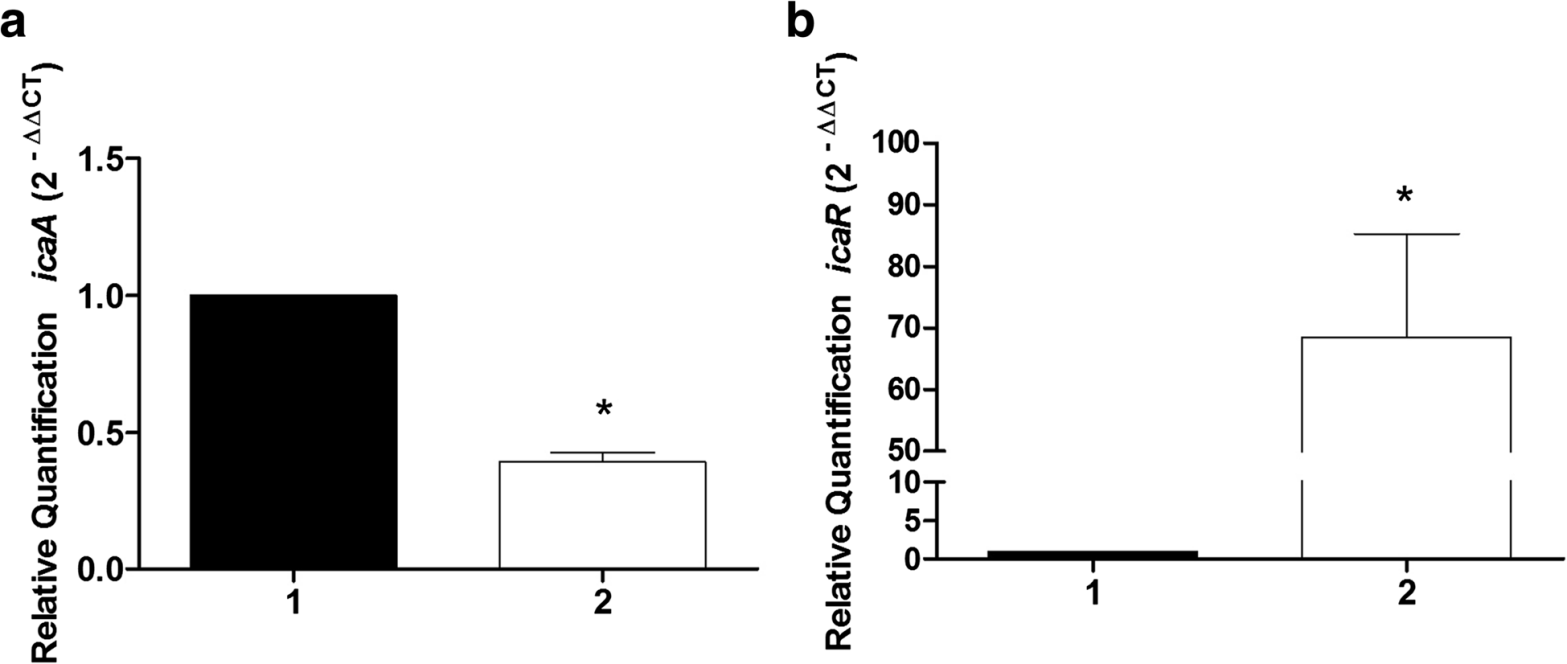
Real-time quantification of *icaA* and *icaR* expression in *S. aureus* CCMB262 treated or not with *L. fermentum* TCUESC01 supernatant. **a** Relative quantification (RQ) of *icaA*. **b** RQ of *icaR*. Treatments were as follows: 1, untreated control; 2, 50 % of the MIC. Values correspond to RQs (2^-∆∆CT^) normalized to*16S rRNA*.*, statistical significance at *p* < 0.05, *t*-test

PIA synthesis depends on the *icaADBC* locus [7]. Thus, we sought to verify whether the phenotypic modulation of *S. aureus* biofilm by *L. fermentum* TCUESC01 was related to the genetic regulation of *icaA* and *icaR* genes. We observed that treatment with *L. fermentum* supernatant (50 % of the MIC) significantly increased the expression of *S. aureus* regulator gene *icaR*. Accordingly, RQ increased from 1 (untreated) to 68.45 (treated) (Fig. 8a), while *icaA* expression dropped from a RQ of 1 (untreated) to 0.39 (treated) (Fig. 8b). These results suggest that the reduction of *S. aureus* CCMB262 biofilm upon treatment with TCUESC01 is associated with inhibition of PIA production.

Biofilms are formed by planktonic cells that bind irreversibly to a solid substrate. Wrapped in a matrix, cells multiply, differentiate, and modify gene expression patterns, in addition to creating channels for nutrient circulation [33]. After ripening, fragments of this bacterial community scatter, carrying all the “mother” community features such as antimicrobial resistance and virulence, and adhere elsewhere to repeat the cycle [34]. When organized into biofilms, bacteria tend to become more pathogenic. Suci et al. (1994) demonstrated the delayed penetration of ciprofloxacin into biofilms of *Pseudomonas aeruginosa* ERC-1 [35]. Seemingly, Ceri et al. (1999) found that the MIC of some antibiotics was around 100 to 1000 times greater for microorganisms in biofilms [36]. These findings underscore the importance of developing new strategies to combat biofilms, such as the use of probiotics. Here, *L. fermentum* TCUESC01 and *L. plantarum* TCUESC02 showed bactericidal properties against *S. aureus* CCMB262. Furthermore, TCUESC01 was able to reduce the formation of *S. aureus* biofilms under subinhibitory conditions. In addition, increased *icaR* and reduced *icaA* expression in *S. aureus* cultures treated with TCUESC01 supernatant indicated that the reduction in biofilm production occurred through modulation of the *ica* operon.

## Conclusions

The results presented in this study suggest an interesting novel application for lactobacilli isolated from fine cocoa. The antimicrobial effect and biofilm inhibition exhibited by the probiotic strain TCUESC01 could be applied to the treatment and prevention of infections with pathogenic bacterial strains.

## Abbreviations

BFI: Biofilm formation index
CCMB: Microorganisms Culture Collection of Bahia
CLSI: Clinical and Laboratory Standards Institute
DAPI: 4′, 6′-diamidino-2-phenylindole
eDNA: Extracellular DNA
Glc: Glucose
MC: Sterility control of the mueller hinton medium
MH: Mueller hinton
MHA: Mueller hinton agar
MIC: Minimum inhibitory concentration
MRS: de Man, Rogosa, and Sharpe
NC: Negative control
PBS: Phosphate-buffered saline
PI: Propidium iodide
PIA: Polysaccharide of intercellular adhesion
RQ: Relative quantification
RT-PCR: Real-time PCR
RZ: Resazurin
SEM: Scanning electron microscopy
TSB: Tryptic soy broth
